# Decision making on vestibular schwannoma treatment: predictions based on machine-learning analysis

**DOI:** 10.1038/s41598-021-97819-x

**Published:** 2021-09-15

**Authors:** Oliver Profant, Zbyněk Bureš, Zuzana Balogová, Jan Betka, Zdeněk Fík, Martin Chovanec, Jan Voráček

**Affiliations:** 1 Faculty of Management, Prague University of Economics and Business, Jindrichuv Hradec, Czech Republic; 2 Department of Auditory Neuroscience, Institute of Experimental Medicine, Czech Academy of Sciences, Prague, Czech Republic; 3 Department of Otorhinolaryngology, 3rd Faculty of Medicine, University Hospital Královské Vinohrady, Charles University in Prague, Prague, Czech Republic; 4 Department of Otorhinolaryngology and Head and Neck Surgery, 1st Faculty of Medicine, University Hospital Motol, Charles University in Prague, Prague, Czech Republic; 5 Department of Cognitive Systems and Neurosciences, Czech Institute of Informatics, Robotics and Cybernetics, Czech Technical University, Jugoslávských partyzánů 1580/3, 160 00 Prague 6, Czech Republic

**Keywords:** Auditory system, Cancer in the nervous system, Neurosurgery, Machine learning

## Abstract

Decision making on the treatment of vestibular schwannoma (VS) is mainly based on the symptoms, tumor size, patient’s preference, and experience of the medical team. Here we provide objective tools to support the decision process by answering two questions: can a single checkup predict the need of active treatment?, and which attributes of VS development are important in decision making on active treatment? Using a machine-learning analysis of medical records of 93 patients, the objectives were addressed using two classification tasks: a time-independent case-based reasoning (CBR), where each medical record was treated as independent, and a personalized dynamic analysis (PDA), during which we analyzed the individual development of each patient’s state in time. Using the CBR method we found that Koos classification of tumor size, speech reception threshold, and pure tone audiometry, collectively predict the need for active treatment with approximately 90% accuracy; in the PDA task, only the increase of Koos classification and VS size were sufficient. Our results indicate that VS treatment may be reliably predicted using only a small set of basic parameters, even without the knowledge of individual development, which may help to simplify VS treatment strategies, reduce the number of examinations, and increase cause effectiveness.

## Introduction

Vestibular schwannoma (VS) is the most common tumor of the temporal bone. It is a benign, mostly solitary and slowly growing tumor that grows from the Schwann cells of the vestibular portion of the 8th cranial nerve. VS causes approximately 80% of the tumors of the pontocerebellar angle, and around 8–10% of intracranial tumors^1^. The symptomatology of VS is mainly caused by the compression or destruction of the surrounding structures, and an obstruction in the flow of cerebrospinal fluid, and comprise mainly asymmetric hearing loss^2,3^, unilateral tinnitus^4^, or balance disorders and cefalea^5^.

Basically, there are two possible approaches to a patient with a VS: a wait-and-scan (WaS) strategy during which the patient undergoes regular checkups with no active treatment, and an active treatment of the tumor. A long WaS monitoring might eventually lead to an increased tumor size and subsequent complicated operation; however if there is no VS progress, such conservative treatment is economic and harmless to the patient. The active treatment (surgery or radiotherapy) is more beneficial in smaller tumors^6^. Although there is always a chance that the tumor will not grow and no intervention would be necessary, the length of postponement of active intervention (even with relatively small tumor growth) can worsen the results^7–9^. Therefore, an untimely decision on active treatment might lead to poorer results and unnecessary costs.

At the initial diagnosis and during the subsequent regular checkups, a number of diagnostic variables is gathered. Based on these variables and their dynamics, a decision on further treatment is made. However, contributions of the individual variables to the final decision may vary; furthermore, for some variables the static values are important, while for other variables the dynamic change is the key. Knowledge of these principles is important in two ways: it could optimize the diagnostic routine by eliminating the unnecessary procedures, and it could support the medical teams in their decisions by providing an objective reasoning of the patient’s state.

Machine learning techniques represent a promising tool for supporting decisions in many disciplines. Statistical processing seeks quantitative identification and an explanation of relationships among variables, however, the precision and reliability of the statistical description strongly depends on a priori assumptions and the size of the data sample. This is particularly limiting when it comes to multidimensional data. The approach of artificial intelligence (to which the machine learning belongs) can overcome these limitations by building a model using known training data, which is subsequently validated using validation data. This model is then utilizable for making predictions or decisions; its performance (correctness of its decisions or predictions) can be further assessed by testing data that were not available to the model during the learning phase.

**Figure 1:**
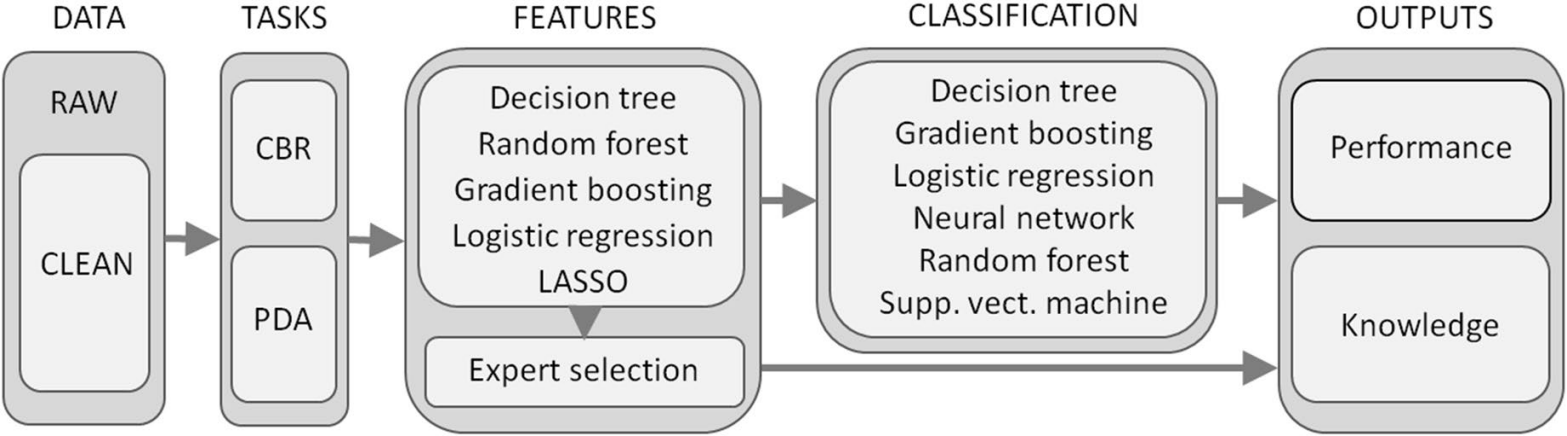
An outsketch of the methodological process used in the analysis. After cleaning the data, the problem was solved in two parallel tasks (CBR and PDA). Using several feature selection methods followed by expert evaluation, the most important predictors of active VS treatment were identified. The identified set of predictors was processed by several classification methods to create models capable of predicting the active VS treatment based on the predictor values. The performance of the models was analyzed using various performance metrics.

The aim of this study was to address the following questions: (1) can a single examination (for example, the initial checkup at the time of VS diagnosis) reliably predict the need for active treatment? (2) If so, what are the diagnostic variables and their values that can lead to such a prediction? (3) When evaluating the dynamics of the patient’s state, which changes of which variables are the most important ones for the decision on further treatment? We address these issues using machine-learning methods of data classification^10^, which is a promising analytical tool particularly in situations when the classical statistical processing is not suitable, e.g., due to extensive data dimensionality, insufficient size of the data sample, or when the necessary a priori assumptions are not met. We approach the problem from two viewpoints. First, we treat each checkup record as an independent entity and analyze which checkups resulted in a decision of active treatment (the so called case-based reasoning, CBR). Second, we take into account the dynamic changes of all the diagnostic variables of each patient and look for those dynamic changes that best characterize the actively treated patients (the so called personalized dynamic analysis, PDA). Data sets for both problems were processed with supervised machine learning methods to identify and justify the most reliable predictors of VS treatment. In both tasks, we seek the minimum set of variables (features) along with their values (static or dynamic), that lead to the most reliable prediction of active treatment. As a result, we present for each task a black-box automated classifier that predicts the active treatment when provided with the appropriate data, and also a transparent set of rules based on a decision tree. An overview of the methodology is shown in Fig. 1. It is important to note that our conclusions were derived entirely from a strictly cleaned data set, which contained no subjective or methodological assumptions that could possibly affect the discovered information. Such unbiased resulting structures can serve as a ground truth, either for subsequent expert evaluations or for the comparison of results with more knowledge-intensive approaches, including statistics.

## Methods

We present the results of a semi-supervised analysis of 388 medical records, characterizing the wait-and-scan (WaS) phase of vestibular schwannoma development for 93 individually followed VS patients. Our group of patients was selected from approximately 400 patients with diagnosed VS, examined at the Department of Otorhinolaryngology and Head and Neck Surgery, 1st Faculty of Medicine, Charles University, University Hospital Motol between 2012 and 2018. The main input criterion was the selection for the WaS protocol based on the initial examination. The original set of source diagnostic variables was cleaned and restructured.

### Data acquisition

Diagnostic data were obtained at the Department of Otorhinolaryngology and Head and Neck Surgery, 1st Faculty of Medicine, Charles University, University Hospital Motol, between 2012 and 2018. The examination procedures, and the informed consent, were approved by the Ethics Committee of the University Hospital Motol, in Prague. All the participants provided their written informed consent to participate in this study; signed written consents are stored at the Department. All procedures were performed in accordance with relevant guidelines and regulations and with the Declaration of Helsinki.

### Data characteristics

The original data set included 388 records of 93 patients (55 females, 38 males; age median 59 years, 44 left-sided VS, 49 right-sided VS). For the 53 patients who were retained in the wait-and-scan regime, the median duration of the overall investigation period was 51 months inclusive of 5 checkups. Within the actively treated group, the median duration of the wait-and-scan regime lasted 37 months and required 3 checkups.

The raw data obtained by commonly used diagnostic techniques were organized in a table, where each row represented a single diagnostic checkup which either resulted in active treatment or not, and where columns corresponded to diagnostic variables as follows:

- Pure tone audiometry [PTA (dB)]—pure-tone hearing thresholds measured separately for each ear at eight frequencies from 0.25 to 8 kHz in attenuated chamber,
- Speech audiometry (measured in the diseased ear in attenuated chamber)—Speech reception threshold [SRT (dB)], Speech discrimination score [SDS (%)], Maximum discrimination level [MDL (dB)], and Maximum discrimination ratio [MDR (%)],
- Magnetic resonance imaging-based descriptors (the size was evaluated on T2-weighted MRI):
  o Size of VS: maximal 1D size [mm],
  o Koos grading (Class 1–4).
- Derived row-based metrics for CBR, calculated from PTAs separately for each ear, and separately for two frequency ranges (full—whole set of frequencies up to 8 kHz, and basic—only frequencies up to 4 kHz):
  o average PTA in dB, denoted as PTA_*X*_AR*n* where *X* can either be VS (diseased ear) or H (healthy ear), and *n* is either 8 (full range) or 4 (basic range); for example, PTA_VS_AR8 is the average PTA of the diseased ear computed from frequencies up to 8 kHz,
  o slope and intercept of linear fit of pure-tone thresholds in dB, denoted as PTA_*X*_SR*n* and PTA_*X*_IR*n*, respectively
  o difference of average PTA between the two ears, denoted as PTA_D_AR4 or PTA_D_AR8.
  o The resultant data set had 184 38-dimensional records.
- Derived column-based metrics for PDA, calculated from time-dependent changes of selected variables (including the row-based ones) separately for each patient:
  o average, denoted as *var*_AC, where *var* is the variable from which the column-based average is computed,
  o slope, denoted as *var*_SC, for example PTA_D_AR4_SC stands for time-dependent slope of the inter-ear difference of average PTA computed over the basic frequency range,
  o intercept, denoted as *var*_IC,
  o last and total differences, denoted as *var*_LC and *var*_TD, respectively.
  o The resultant data set had 42 24-dimensional records.

Several other functions were examined in the patients (auditory brainstem response (ABR), otoacoustic emissions (OAE), vestibular function), however, they were either not recordable (ABR, OAE) or were not consistently provided over the course of time, therefore they were excluded from the current analysis.

Subjective characteristics of the patients, such as vertigo or tinnitus, were also gathered but were not included in the current analyses. The current study was designed as entirely non-parametric and data-driven; therefore to avoid any possible subjectivity we purposely suppressed the influence of non-deterministic factors, including the patients' subjective characteristics. For the same reason, all incomplete records were removed instead of artificially imputing the missing values. Additionally the phase of data transformation was omitted, as it usually leads to the normalization or equalization of data distributions. Although our restrictions caused the loss of some information, this approach avoids unjustified biases, is fully repeatable and extendable, and as such represents a core baseline model, which can later serve as a reliable benchmarking etalon for comparison with alternative ways of processing; namely including traditional parametric statistical techniques.

### Data processing—general

The applied methodology follows the general Knowledge Discovery in Databases process, introduced in^11^ or^12^. The data were processed with supervised, internally transparent machine learning methods as follows:

- No a priori assumptions concerning the cumulative characteristics of data were made, so the presented results are not biased by any artificial modifications, like imputations or transformations.
- Only complete records were selected for further processing.
- Two complementary approaches: (1) static, anonymized CBR, and (2) personalized PDA, were applied to discover knowledge hidden in a multi-dimensional space.
- CBR assigns single medical records (rows) to the binary target decisions on the treatment (WaS/active), it considers neither the characteristics of individual patients, nor their history of VS progress.
- PDA also performs binary classification, but works with the temporal courses of selected variables taken from the complete WaS checkup history of single patients. Thus, every processed sample summarizes the complete column-wise WaS history of the given patient.
- An interactive reduction of dimensionality (feature selection) preserving the meaning and relations among the original variables was performed, to exclude the less significant features, and simplify the problem and increase the generalization capabilities of the resulting structures.
- The data for all supervised learning tasks were equally balanced with respect to the target, and randomly divided into the training, validation, and test sets in the proportion 50:30:20. The first two partitions were used for learning and optimization of the desired type of discrimination function, the last subset contained unseen data and served for the numeric evaluation of classification performance.

### The supervised elimination of redundant features

An initial reduction of dimensionality was performed in all classification tasks, using the five below-listed techniques implemented with the StatExplore, HP Random Forest, Gradient Boosting, Variable Selection and HP Variable Selection nodes of SAS Enterprise Miner:

1. Decision or classification tree^13–15^ with Chi-square split^16^.
2. Random forest^17,18^ with the Gini impurity index *G*^19,20^ as the node splitting metrics.
3. Gradient boosting^21,22^ using Gini impurity index for updating the decision tree.
4. Logistic regression^23–25^ with respect to the target class, applied to the results of forward stepwise regression^26^ of a gradually reduced set of pairwise (R-squared) correlations^27^.
5. Least absolute shrinkage and selection operator (LASSO)^28,29^.

In addition to these algorithms, an expert (manual) selection of the most significant features was performed, which is also the main output from the knowledge elicitation phase. At the end of this iterative process, we proposed the minimal set of variables efficiently characterizing the analyzed problem, based on the outputs of the previous five algorithmic methods. The primary criterion for selection of a given variable was its occurrence among the best ten candidates, which must be either greater or equal to 3, or its average ranking lower or equal to 5. Perspective combinations of such preliminarily selected candidates were interactively analyzed, to eliminate the least significant members and maximize the credibility of the discovered knowledge.

### Supervised learning and classification

In the classification stage we used the following techniques:

1. Decision tree, random forest, gradient boosting and logistic regression, all referred to in the previous section.
2. Support vector machine with radial basis function kernel^30–32^.
3. Feed-forward neural network^33,34^.

The optimal classifier was selected as the best performing combination of the six feature selection techniques given in the previous section (logistic regression, decision tree, random forest, gradient boosting, LASSO, and interactive expert selection) with the six types of classifiers given here.

### Performance metrics

To evaluate classification performance, several indicators were used:

- Accuracy (ACC)—the rate of correct classification for the evaluated data set:
  $$ACC= \frac{TP+TN}{TP+TN+FP+FN}= \frac{TP+TN}{P+N}$$

where *TP* is the true positive, *TN* is the true negative, *FP* is the false positive, *FN* is the false negative, *P* is the all real positive (*P* = *TP* + *FN*), *N* is the all real negative (*N* = *TN* + *FP*) cases.

- Sensitivity (also recall or true positive rate, *TPR*)—the ability to correctly classify TP cases:
  $$TPR= \frac{TP}{TP+FN}= \frac{TP}{P}$$
- Specificity (also selectivity or true negative rate, *TNR*)—the ability to correctly classify TN cases:
  $$TNR= \frac{TN}{TN+FP}= \frac{TN}{N}$$
- Precision (also positive predictive value, *PPV*)—the rate that the predicted positive is TP:
  $$PPV= \frac{TP}{TP+FP}$$
- Area under the Receiver operating characteristic curve (AUC)^35,36^. Practically applicable classifiers should have AUC > 0.6, while AUC > 0.9 indicates an excellent performance.
- Average square error (ASE)—squared metric difference between the target and continuous output of the discrimination function, divided by the number of samples.

## Results

**Figure 2:**
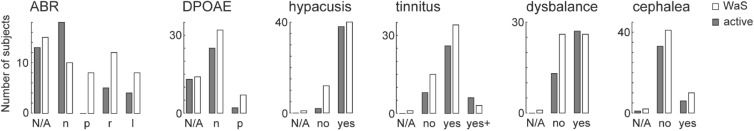
Diagnostic data of the patients included in the analysis. The bars represent the number of subjects having a certain characteristic. *N/A* not available; *n *ABR/DPOAE response not present; *p* ABR/DPOAE response present; *r* ABR with signs of retrocochlear lesion; *l* ABR with prolonged latencies. *Yes +*  annoying tinnitus. Gray bars—actively treated patients; white bars—wait-and-scan patients.

**Figure 3:**
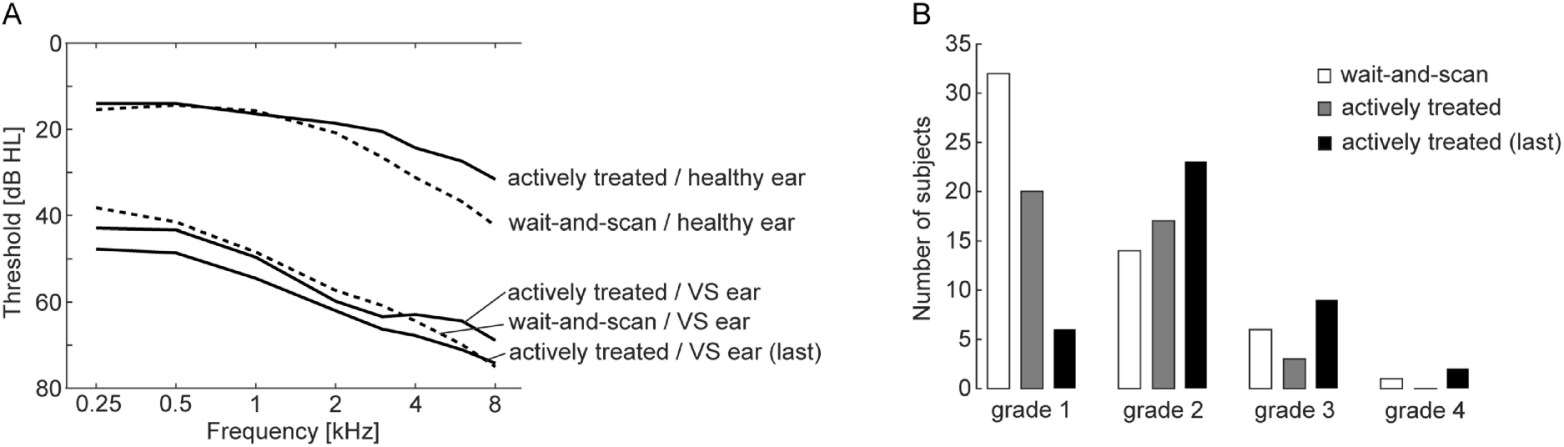
Hearing thresholds and tumor sizes of the patients included in the analysis. **(A)** Average audiograms recorded in the healthy and diseased ears in wait-and-scan patients and in the patients later changed to active treatment during the initial examination, plus the average audiogram of the diseased ear in the actively treated subjects recorded immediately before the change from wait-and-scan to active treatment. **(B)** Histogram of Koos grades identified in the actively treated and wait-and-scan patients during the initial examination, and in the actively treated subjects recorded immediately before the change from wait-and-scan to active treatment, the bars represent numbers of subjects having a certain Koos grade.

The general diagnostic data of the patients included in the analysis are illustrated in Fig. 2. These graphs show the number of subjects having a certain result of ABR and distortion products of OAE (DPOAE) examinations, as well as subjective characteristics such as hypacusis or tinnitus. Figure 3A depicts averaged audiograms recorded from both healthy and VS ears during the initial examination, plus the average audiogram of the diseased ears recorded immediately before the change from wait-and-scan to active treatment. Figure 3B shows the histogram of Koos grades recorded during the initial examination in wait-and-scan patients, patients who were later changed to active treatment, and in the actively treated patients recorded immediately before the change from wait-and-scan to active treatment.

The section below summarizes the results of the two interrelated analytic phases, dimensionality reduction including knowledge extraction, and supervised learning for both CBR and PDA experiments.

### CBR—dimensionality reduction and knowledge extraction

The output of this method is a set of the most important diagnostic characteristics (variables) along with their significant values. The method aims to provide a transparent set of rules which, using the values of the selected variables, can simply be used generally to support the decision on VS treatment.

Initially, the dimensionality of the full set of CBR variables was reduced with five algorithmic methods (see Table 1). Each of the methods provided 10 variables, rated as the most important for the prediction of VS treatment. Using the variables suggested by the algorithmic methods we manually performed an expert ranking, resulting in an initial version of a reduced set of variables (denoted as CBR_EXP_INI). By interactive minimizations of this initial set we finally proposed a minimum set of variables (CBR_EXP_FIN), necessary for the reliable prediction of VS treatment. Table 2 shows the performance for different sets of variables; it is obvious that the removal of unnecessary variables actually improves the prediction accuracy, and furthermore, the output generated by the expertly found features is comparable with the average performance of the three best automated supervised classifiers and feature selectors marked as CBR_CLASS_ (see Tables 5 and 6 in the next section). In addition, Table 2 presents the quality of adaptation on known samples (an average of performance on training and validation data).

**Table 1:** Predictors, extracted from CBR data, ordered according to their significance for applied dimensionality reduction method.

Ord	Decision tree	Random forest	Gradient boosting	Logistic regression	LASSO	Expert selection (CBR_EXP_)			
						Initial	Num	Avg	Final
1	PTA_VS_SR8	Koos	Koos	Koos	Koos	Koos	5	1.2	Koos
2	Koos	PTA_VS_SR8	PTA_VS_SR8	Size	PTA_H_SR8	SRT	5	3.8	SRT
3	PTA_D_AR4	SRT	PTA_H_SR8	SRT	Size	PTA_VS_SR8	4	3.3	PTA_VS_SR8
4	SRT	PTA_VS_AR4	SRT	PTA_VS_0.25	PTA_VS_SR4	PTA_H_SR8	4	4.0	PTA_H_SR8
5	PTA_H_SR8	PTA_VS_3	Size	PTA_H_8	SRT	Size	4	5.0	-
6	PTA_VS_SR4	PTA_H_SR8	PTA_VS_IR8	PTA_H_0.5	PTA_H_IR8	PTA_VS_SR4	3	5.7	-
7	PTA_D_AR8	PTA_VS_SR4	PTA_D_AR4	PTA_H_2	SDS	PTA_VS_0.25	3	7.0	-
8	PTA_H_IR8	PTA_VS_0.25	PTA_D_AR8	PTA_VS_6	PTA_VS_SR8	PTA_H_IR8	3	7.7	-
9	PTA_VS_3	MDL	PTA_H_IR8	PTA_VS_8	PTA_VS_0.25	PTA_D_AR4	2	5.0	PTA_D_AR4
10	Size	PTA_H_8	PTA_VS_1	SDS	MDR	-	-	-	-

Irrelevant variables were rejected from expert selection. Variables and metrics are expressed as follows (see “Methods”): (a) suffix 4 holds for the basic frequency range, suffix 8 holds for the full frequency range; (b) subscripts H and VS hold for the healthy or diseased ear, respectively, subscript D holds for the difference of averaged PTA values between the two ears; (c) tailing abbreviations have the following meaning: *AR* average row wise, *IR* intercept row wise, *SR* slope row wise.

**Table 2:** Performance of gradually reduced expert set of variables for CBR data.

Variables		Train and validation (AVG, %)						Test (%)					
Set	Number	ACC	PPV	TPR	TNR	AUC	ASE	ACC	PPV	TPR	TNR	AUC	ASE
CBR	38	78	75	84	72	87	15	72	69	79	65	81	19
CBR_EXP_INI	9	82	79	88	76	84	15	76	72	83	69	76	19
CBR_EXP_FIN	5	84	81	90	78	88	14	81	78	88	74	78	17
CBR_CLASS_	38	86	84	91	82	92	11	88	85	93	82	87	12

Based on the aforementioned findings, we can claim that knowledge of the Koos classification, SRT, and three PTA-derived variables, provides sufficient information for a reliable VS surgery decision; even in the case of a single medical checkup. Therefore it may be feasible to exclude clinical tests of the less significant features, which can make the daily diagnostic routine faster and cheaper.

**Figure 4:**
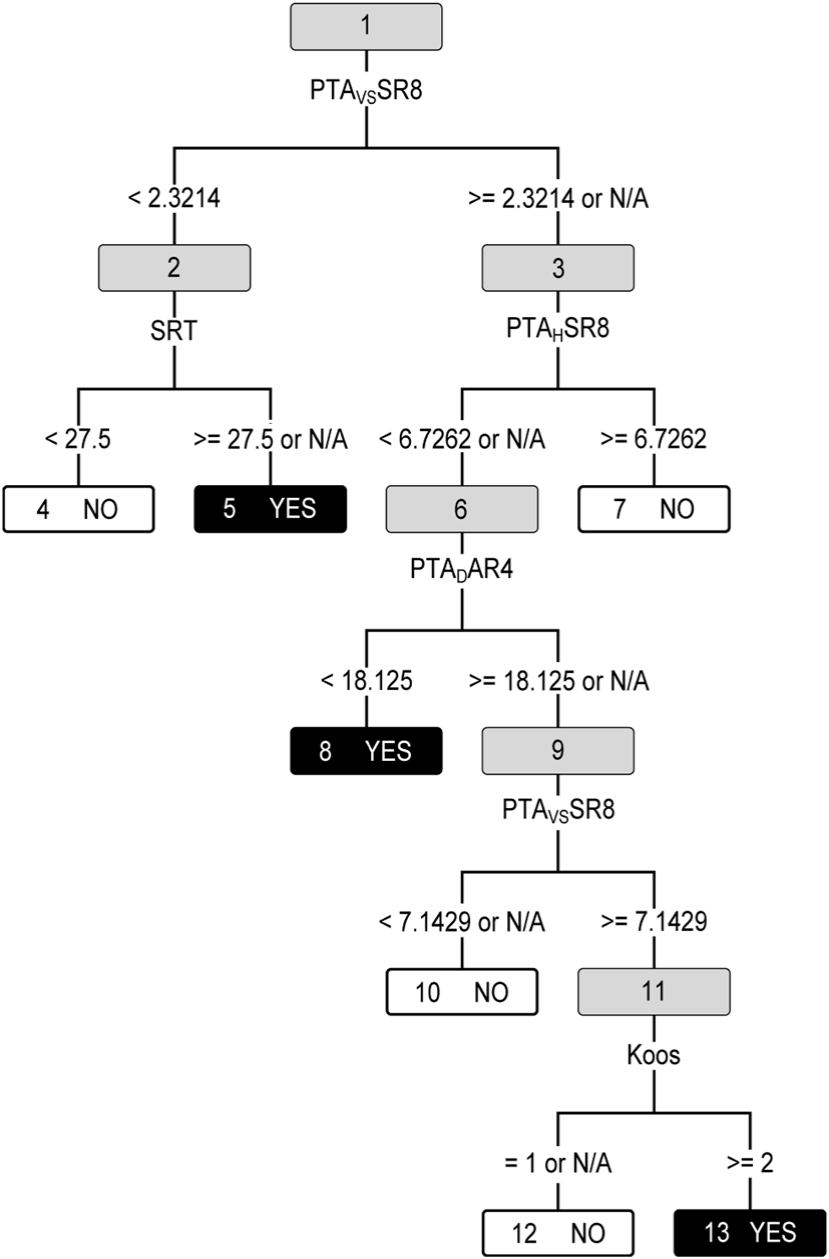
CBR decision tree. A decision tree for CBR_EXP_FIN variables, applied on CBR data.

Using the individual variable values, it is now possible to decide whether to perform active treatment (*Yes* decision) or not (*No* decision). An important question is what the boundary values of the variables are, i.e., at which level each variable switches the decision from No to Yes. The answer, however, is not unique because the selected features can be assigned into numerous structurally different solutions with comparable performances. One possible solution is given in Fig. 4, and in detail in Table 3. By traversing this binary decision tree according to the rules, we finally arrive at the decision in the leaves; decision accuracy in the leaf nodes is approximately 80%. The ability of the decision tree to also handle missing (N/A) values is yet another advantage of this technique. An example of several CBR records taken from our data and the corresponding decisions is shown in Table 4.

**Table 3:** Tabular representation of a decision tree for CBR_EXP_FIN variables, applied on CBR data.

Node ID	Samples	Node rule	No	Yes
1	184	PTA_VS_SR8 ≥ 2,3 or N/A	2	3
2	61	SRT ≥ 27.5 or N/A	4	5
3	123	PTA_H_SR8 ≥ 6.7	6	7
4	9	No		
5	52	Yes		
6	86	PTA_D_AR4 ≥ 18.1 or N/A	8	9
7	37	No		
8	17	Yes		
9	69	PTA_VS_SR8 ≥ 7.1	10	11
10	39	No		
11	30	Koos = 2	12	13
12	18	No		
13	12	Yes

**Table 4:** Inferences for selected sample CBR records using a decision tree learned from CBR_EXP_FIN variables.

ID	Koos	SRT	PTA_VS_SR8	PTA_H_SR8	PTA_D_AR4	Real target	Nodes visited	Prediction
1	1	57	11.3	1.3	28.8	No	1, 3, 6, 9, 11, 12	No
2	1	30	4.7	0.2	5.0	Yes	1, 3, 6, 8	Yes
3	2	110	10.7	7.6	43.8	No	1, 3, 7	No
4	2	37	2.1	0.1	16.3	Yes	1, 2, 5	Yes
5	3	60	4.6	9.8	17.5	No	1, 3, 7	No
6	3	110	2.1	3.3	26.3	Yes	1, 2, 5	Yes
7	4	110	5.2	2.3	68.8	No	1, 3, 6, 9, 10	No
8	4	110	0	3.0	98.8	Yes	1, 2, 5	Yes

These experimental results confirmed the applicability of the variable set CBR_EXP_FIN for the reliable predictions of VS surgery. The presented structural representation (i.e., the decision tree in Fig. 4) can help practitioners in a more informed analysis of diagnostic results.

### CBR—supervised learning

The previous method gave a transparent set of significant variables and their values that can be directly used for the prediction or decision on VS treatment. However, its result is generally ambiguous; furthermore, our intention to minimize the variable set as much as possible might lead to a certain loss of accuracy. For such reasons, we also decided to create a black-box-like solution based on an automated feature selector followed by a classifier. We identified and parametrized perspective combinations of the six feature selectors with six classifiers. As in the previous method, the CBR data set was split into the training, validation, and test partitions, and batch processed for all the 36 combinations of feature selectors and classifiers. The results of classification accuracy are summarized in Table 5.

**Table 5:** Performance of applied combinations of dimensionality reduction and classification techniques on test set for CBR and CBR_EXP_FIN data.

Feature selection method	Classification accuracy (ACC) for test set (%)						
	Decision tree	Rand. Forest	Gradient boosting	Logistic regression	Supp. vect. machine	Neural network	Avg
Decision tree (CBR)	76	79	84	82	79	76	*79*
Random forest (CBR)	76	79	87	76	76	68	*77*
Gradient boosting (CBR)	84	82	86	84	76	89	*84*
Logistic regression (CBR)	67	78	68	86	83	85	*78*
LASSO (CBR)	74	79	79	76	74	82	*77*
Expert (CBR_EXP_FIN)	76	76	87	79	76	74	*78*
Avg	76	79	82	81	77	79	79

Table 5 shows that the gradient boosting algorithm is on average the best performing algorithm for both data processing phases (i.e., it works the best both as a feature selector and as a classifier). The globally best result was generated by its combination with neural network (89%). The performance of the fixed expert selection of variables in the CBR_EXP_FIN set is also remarkable, particularly when followed by a gradient boosting classifier.

**Figure 5:**
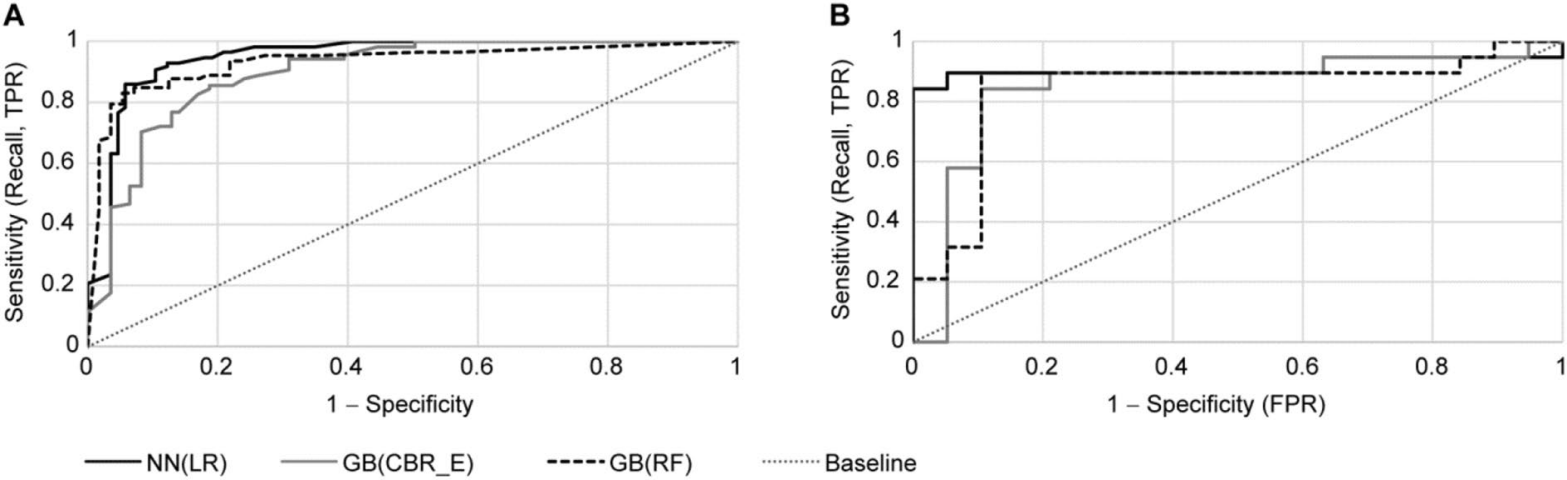
Receiver operating characteristic (ROC) curves of the three best performing classifiers for CBR data. (A) Averaged ROC curves of training and validation sets, **(B)** ROC curves for the test sets.

Full results of the three best performing combinations are shown in Table 6, the corresponding Receiver operating characteristic (ROC) curves are depicted in Fig. 5. The slightly worse performance for the train and validation set, in comparison with the test set, was caused by a larger validation error. However, as the key performance indicator was behavior for unknown test data, we accepted this local decrease which was mainly caused by a small number of learning samples in comparison with the number of significant variables. Regardless, Table 6 shows that the absolute test accuracies, as well as biases and variances of the winning combinations, are sufficient for daily use.

**Table 6:** Detailed metrics for the three best performing classifiers for CBR data.

Classification (feature selection)	Train and validation (avg, %)						Test (%)					
	ACC	PPV	TPR	TNR	AUC	ASE	ACC	PPV	TPR	TNR	AUC	ASE
Neural network (Gradient boosting)	84	82	88	80	93	10	89	87	93	85	92	8
Gradient boosting (CBR_EXP_FIN)	84	81	89	79	89	13	87	84	94	80	86	15
Gradient boosting (random forest)	91	89	96	86	94	10	87	84	93	81	84	14

To compare the results obtained from the traditional, two-stage processing with those obtained from a complementary one-shot algorithm, we processed the full CBR dataset with the Deep learning algorithm. A set of experiments employing this modern technique was performed on a fully connected thee-layered network. The layers included 38, 76, and 2 neurons with the rectified linear activation function. The network was trained with gradient descend back-propagation method. Such paradigm resulted in the following best performance:

$$ACC=82\%, PPV=78\%, TPR=89\%, TNR=75\%,AUC=88\%,ASE=13\%$$

which is slightly worse than performance of classifiers with separate feature selection and classification stages. This result was partially determined by low cardinality of the processed dataset, as the Deep learning approach is suitable particularly for processing of extensive multidimensional datasets.

### PDA—dimensionality reduction and knowledge extraction

While the CBR data set and the corresponding methods generated their predictions based only on a single medical checkup, the PDA data set takes into account the individual history of checkups for each patient. It is evident that the time-dependent development of diagnostic variable values may bring important information into the decision process. Therefore, we also repeated the same ranking and specification procedures described for the CBR data set for the PDA data set, in order to minimize the number of input variables and to obtain a transparent set of decision rules. The variables suggested by the feature selectors and the structure of the resulting expert set (PDA_EXP_) are shown in Table 7. Table 8 shows the detailed performance metrics of the gradually optimized variable set. As with the CBR data set, in this case we also see the positive effect of the lower number of inputs on the overall performance and primary role of size-oriented VS metrics.

**Table 7:** Predictors, extracted from PDA data set, ordered according to their significance for each dimensionality reduction method.

Ord	Decision tree	Random forest	Gradient boosting	Logistic regression	LASSO	Expert selection (PDA_EXP_)			
						Initial	Num	Avg	Final
1	Size_LD	Size_LD	Koos_LD	Size_LD	Koos_LD	Koos_LD	5	2	Koos_LD
2	Koos_LD	Koos_LD	Size_SC	PTA_VS_AR4_SC	PTA_D_AR4_IC	Size_LD	4	1.5	-
3	Size_AC	Size_SC	Size_LD	Size_SC	SRT_IC	Size_SC	4	4.5	Size_SC
4	PTA_VS_AR4_AC	SRT_LD	PTA_VS_SC4	Koos_LD	SRT_LD	Koos_TD	3	6.7	-
5	Koos_TD	Koos_TD	SRT_SC	PTA_D_AR8_SC	PTA_VS_AR8_IC	PTA_D_AR4_SC	3	7.7	PTA_D_AR4_SC
6	PTA_VS_AR8_AC	PTA_VS_AR8_IC	PTA_VS_AR8_SC	PTA_VS_AR8_SC	PTA_D_AR8_LD	PTA_VS_AR4_SC	3	5.3	PTA_VS_AR4_SC
7	PTA_D_AR8_IC	PTA_D_AR4_SC	PTA_VS_AR4_LD	PTA_VS_AR8_SC	PTA_D_AR8_IC	PTA_D_AR8_IC	3	7.7	–
8	PTA_D_AR4_SC	PTA_D_AR4_LD	PTA_D_AR8_SC	PTA_D_AR4_SC	PTA_VS_AR4_IC	Size_AC	3	7.3	–
9	SRT_AC	PTA_D_AR8_AC	PTA_D_AR8_LD	PTA_D_AR8_IC	Size_AC	–	–	–	–
10	Size_SC	PTA_VS_AR4_SC	Size_AC	Koos_TD	PTA_VS_AR4_AC	–	–	–	–

Irrelevant variables were rejected from expert selection. Variables and metrics are expressed as follows (see “Methods”): (a) suffix 4 holds for the basic frequency range, suffix 8 holds for the full frequency range; (b) subscripts H and VS hold for the healthy or diseased ear, respectively, subscript D holds for the difference of averaged PTA values between the two ears; (c) tailing abbreviations have the following meaning: *AR* average row wise, *IR* intercept row wise, *SR* slope row wise, *AC* average column wise, *IC* intercept column wise, *SC* slope column wise, *LD* last difference, *TD* total difference.

**Table 8:** Performance of gradually reduced set of variables for the PDA data set.

Variables		Train and validation (Avg, %)						Test (%)					
Set	Number	ACC	PPV	TPR	TNR	AUC	ASE	ACC	PPV	TPR	TNR	AUC	ASE
PDA	24	87	84	92	82	88	12	82	79	89	75	81	16
PDA_EXP_INI	8	87	84	92	82	88	12	82	79	89	75	81	16
PDA_EXP_FIN	4	87	85	92	82	90	11	82	79	89	75	81	16
PDA_CLASS_	24	85	82	91	79	83	14	90	88	95	86	100	10

**Figure 6:**
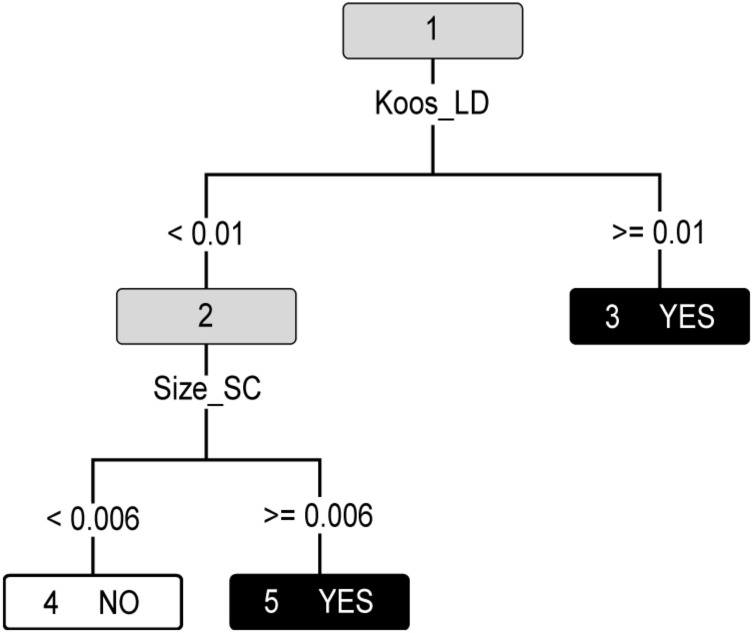
PDA decision tree. A decision tree for PDA_EXP_FIN variables, applied on PDA data.

The decision tree constructed from the PDA_EXP_FIN variable set naturally suppressed both the PTA-related indicators, as is shown in Fig. 6 and Table 9. The result can be simply interpreted: if there is any change in Koos classification from the previous checkup, surgery is recommended. If the Koos class remains unchanged, the Size growth is checked and if the trend is positive, surgery is indicated. Generally, both identified variables are so significant that no other diagnostic procedures are necessary (neither the expertly identified PTA). Regardless, if they were performed, the results can enhance the existing CBR knowledge base.

**Table 9:** Tabular representation of a decision tree for PDA_EXP_FIN data set.

Node ID	Samples	Node rule	Yes	No
1	42	Koos_LD < 0.01	2	3
2	31	Size_SC < 0.006	4	5
3	11	Yes		
4	24	No		
5	7	Yes		

### PDA—supervised learning

Supervised PDA experiments suffered from the low number of samples and, consequently, the small size of the test set. Although this fact was efficiently compensated with the inherent dominancy of both the size-related variables, test classification outputs were discretized into several levels, as obvious from Table 10. The overall weaker performance of the interactively selected set of features PDA_EXP_FIN was caused by its fixed and relatively wide structure in comparison with the other dimensionality reduction techniques. In this specific situation, LASSO algorithm demonstrated the best average feature selection capabilities and its main component, logistic regression, as one of the most powerful classification algorithms on a global scale. Such conclusions correspond with the general knowledge concerning the classification of over-determined binary targets^37^, and were also confirmed with the detailed characteristics of the best performing algorithms for the PDA task, presented in Table 11. Accordingly, the PDA data analysis confirmed the statement that the interim growth of VS itself, is the strongest and sufficient predictor of VS surgery.

**Table 10:** Performance of applied combinations of dimensionality reduction and classification techniques on test set for PDA and PDA_EXP_FIN data.

Feature selection method	Classification accuracy (ACC) for test set (%)						
	Decision tree	Rand forest	Gradient boosting	Logistic regression	Supp. vect. machine	Neural network	Avg
Decision tree (PDA)	80	80	80	90	80	70	80
Random forest (PDA)	80	70	80	70	90	70	77
Gradient boosting (PDA	80	80	80	90	80	80	82
Logistic regression (PDA)	80	60	80	80	90	90	80
LASSO (PDA)	90	90	90	90	80	90	88
Expert (PDA_EXP_FIN)	79	79	79	63	63	68	72
Avg	82	77	82	81	81	78	80

**Table 11:** Detailed metrics for three best performing classifiers on PDA data set.

Classification (feature selection)	Train and validation (avg, %)						Test (%)					
	ACC	PPV	TPR	TNR	AUC	ASE	ACC	PPV	TPR	TNR	AUC	ASE
Neural network (logistic regression)	85	82	91	79	88	14	90	88	95	86	100	10
Logistic regression (decision tree)	85	82	91	79	81	14	90	88	95	86	100	10
Logistic regression (gradient boosting)	85	82	91	79	80	15	90	88	95	86	100	10

## Discussion

Over recent years, several studies have addressed the possibility of predicting VS growth, or a change from a conservative to an active treatment^38–48^. Their outcomes are, however, ambiguous; some studies are inconclusive or fail to find any significant predictor of VS growth^38,45^. The majority of the previous results state that the tumor size and also the degree of vestibular disorder are the key variables which influence the switch from conservative to active treatment. The above mentioned studies mostly analyzed the individual progress of symptoms, i.e., they worked in a manner similar to our PDA. Two studies specifically tested the hypothesis that VS growth could be predicted by the available data at diagnosis (i.e., the approach similar to our CBR); the study of Herwadker et al.^49^ found no significant predictors, while Wolbers et al.^50^ identified the long duration of hearing loss and intracanalicular localization of the tumor as the main predictors of a non-growing VS.

Here we present a novel approach to this issue which uses semi-supervised machine-learning techniques to create, parametrize, and evaluate four different models for the prediction of active treatment of vestibular schwannoma:

1. CBR—prediction from static variables
  - automated black-box classifier providing predictions given the input data
  - transparent set of rules (a decision tree) to support the decision on VS treatment
2. PDA—prediction from dynamic variables
  - automated black-box classifier providing predictions given the input data
  - transparent set of rules (a decision tree) to support the decision on VS treatment

The models were trained, validated, and tested using different subsets of the source data, which means that their performances (accuracy etc.) represent realistic values obtained with unknown data. In the applied methods, we concentrated on preservation of the original meaning of the individual attributes so that they remain transparent and interpretable during the entire classification process. This means that we used no multiplicative or other nonlinear transformations, but we instead employed only generalized linear models (LASSO, logistic regression, decision tree) and generalized (random) additive models, represented with the gradient boosting and random forest approaches. Although the latter two approaches are internally non-transparent, they still work with the original meaning of attributes.

The major findings state that using a simple decision tree it is possible to predict VS treatment, even from the static values of a few basic variables (Koos classification, speech reception threshold, and pure tone audiometry), with approximately 80% accuracy. Ultimately a higher accuracy (89%) can be achieved using a black-box classifier on the static data. From the dynamic point of view, we found that VS treatment can be predicted using dynamics of solely size-oriented variables (Koos classification and 1D size), both with a decision tree and with the black-box classifier. The prediction accuracy is slightly higher than that of the CBR approach.

Besides the provided prediction mechanisms alone, our analyses also indicate that only pure-tone hearing thresholds in both ears, speech reception threshold in the diseased ear, and Koos classification, are necessary at the first checkup (these variables are used in the static predictions); while during the subsequent follow-up, mainly the size-derived metrics and their dynamics play a role in the decision process. These findings might help to make the procedures related to the monitoring and treatment of VS patients more time- and cost-efficient, by eliminating the unnecessary measurements.

### Supervised feature selection

The selection of the most important variables is essential in classification tasks where the number of available samples is comparable with the number of input variables, as over-fitted structures are characterized by the poor classification of unknown samples and low generalization ability^51–54^. Considering that both CBR and PDA tasks belong to this category, an initial reduction of dimensionality was unavoidable. Employing the outputs of five dimensionality reduction techniques, we manually performed an expert selection of the most significant features. We believe that the final selection, numerically over-performing the initial configuration, optimally characterizes the key diagnostic symptoms, based on which the reliable VS surgery decision can be made at the very earliest.

### Supervised learning and classification

The supervision in learning lies in the fact that the searched discrimination function is built from samples with a-priori known output membership. In contrast to the dimensionality reduction, internal interpretability of the learned classifier is not required, which results in a black-box-like nature. The previously introduced tree and regression-based techniques were re-used for the selection of significant variables but, as opposed to the manual interpretation of their results, performed in the feature selection process; this first stage was followed here by a learned classification algorithm.

The main mission of the classification task is the best performing inference, i.e. an accurate assignment of real-world clinical data to the predefined classes (in our case, wait-and-scan versus active treatment). Such black-box-like solutions are widely accepted in practice nowadays, especially in connection with deep learning applications^55^. Moreover, the user can still interact, even with the nontransparent classifies, and analyze their responses by manually adjusted inputs. An optimal classifier was selected as the best performing combination of a feature selection technique with a learned classifier. For the CBR data, it was found to be the combination of gradient boosting and a neural network; in the case of the PDA data set, the optimal performance was achieved using combinations of a logistic regression/neural network, or decision tree/logistic regression, or gradient boosting/logistic regression.

### Potential limitations of our study and future directions

We are aware of the potential limitations of our study. Firstly, although we have assembled a relatively large amount of data from our participants, the final cleaned set contained a smaller number of records due to inconsistency in examination over the years (especially in the cases of ABR and OAE, as they were often not present during the initial examination), and unavailability of some variables in some of the records. A lower number of records may cause a decreased performance of the model, yet it avoids biases resulting from the usage of incomplete or potentially incorrect data. In the current analyses, we primarily focused on audiometric data, although information about potential vestibular pathology could be added to the decision making process in the future. Secondly, we omitted the patients’ subjective input to avoid any subjectivity in the data set; however, our clinical experience shows that the subjective worsening of symptoms (that does not necessarily match the objective measurements) might be a strong factor influencing the decision about further VS treatment. Thirdly, our approach to the VS treatment is not purely based on objective measures, but also on the patients’ preference and expectations, and also on the surgeons’ experience and skill level; therefore the presented model is not expected to replace those inputs, but to support the decision making in deciding whether to directly opt for surgery or wait and scan.

Based on our results the future perspectives of our research using the supervised machine learning approach will be the inclusion of not only audiometric but also the vestibular data from our subjects, which would lead to an even more complex prediction model of the VS behavior. The conclusions formulated from supervised learning will be further enhanced with unsupervised analyses, including the linear and nonlinear clustering of data and variables, applied to the full-dimensional data set.

## Conclusions

Using semi-supervised machine-learning algorithms complemented with expert (manual) interactive analyses, we developed practical tools to support the decision process related to the treatment of vestibular schwannomas. These tools comprise of simple decision rules (decision trees) for both static and dynamic data offering accuracy of around 80%, and automated black-box classifiers offering even better performance. Our results already indicate that from the initial data obtained at diagnosis (size of the tumor (Koos classification and 1D size in T2 weighted MRI), speech perception (described by SRT) and pure tone average), it is possible to predict the need of VS active treatment. Furthermore, we propose minimum sets of diagnostic variables which are crucial for deciding on VS treatment. Overall, these findings can be used to make the diagnostic and decision-making procedures more time-and cost-efficient, by focusing on the important metrics and eliminating the unnecessary measurements.

## Data Availability

Data are available at the authors upon request.
